# Investigation of Basophil Activation Test for Diagnosing Milk and Egg Allergy in Younger Children

**DOI:** 10.3390/jcm9123942

**Published:** 2020-12-05

**Authors:** Yoon Hee Kim, Young Suh Kim, Younhee Park, Soo Yeon Kim, Kyung Won Kim, Hyon Suk Kim, Myung Hyun Sohn

**Affiliations:** 1 Department of Pediatrics, Institute of Allergy, Brain Korea 21 Project for Medical Science, Yonsei University College of Medicine, Seoul 03722, Korea; yhkim@yuhs.ac (Y.H.K.); youngsuh817@gmail.com (Y.S.K.); SOPHI1@yuhs.ac (S.Y.K.); KWKIM@yuhs.ac (K.W.K.); 2 Gangnam Severance Hospital, Seoul 06273, Korea; 3 Severance Hospital, Seoul 03722, Korea; 4 Department of Laboratory Medicine, Yonsei University College of Medicine, Seoul 03722, Korea; YOUNHEEP@yuhs.ac (Y.P.); KIMHS54@yuhs.ac (H.S.K.)

**Keywords:** atopic dermatitis, anaphylaxis, basophil activation test, child, diagnosis, food allergy

## Abstract

In children with concomitant food allergy and atopic dermatitis (AD), uncovering the causative food allergen is more arduous. We evaluated the basophil activation test (BAT) for its diagnostic value in children, including those with AD, for milk or egg allergy. We simultaneously measured serum-specific immunoglobulin E (sIgE) levels and performed BATs for cow’s milk and egg white. We compared their overall diagnostic performance using the area under the receiver operating characteristic curve (AUC) with the Delong method and compared them in children with AD. Analyses were completed for 75 children for milk allergy and for 85 children for egg allergy. The sIgE and percentage of basophils with the expression of CD63 were correlated for both milk (r = 0.384, *p* < 0.001) and egg (r = 0.557, *p* < 0.001). The AUC of sIgE (0.701) for milk allergy was significantly increased when combined with the BAT (0.805; *p* = 0.029). In children with AD, the AUC of the BAT (0.924) for milk allergy was significantly larger than that of sIgE (0.701; *p* = 0.017). The BAT is a potentially useful diagnostic tool for milk allergy in children when combined with sIgE. Moreover, it may be a surrogate marker for milk allergy in children with AD.

## 1. Introduction

The gold standard for the diagnosis of food allergy (FA) is a double-blind, placebo-controlled food challenge [1]. However, it is a time-consuming test and poses a risk of causing an acute allergic reaction, which might be potentially severe [1]. Therefore, in clinical practice, the diagnosis of FA is based on a combination of a history of immediate-type allergic reactions to food and the presence of sensitization to causative food [2]; however, in some equivocal cases, an oral food challenge (OFC) remains necessary. Therefore, there is a need for an accurate FA test that can reduce the need for OFC, changing clinical practice. In addition, with increased awareness of FA among the general population, an accurate and precise diagnosis is important to avoid unnecessary dietary restrictions and anxiety associated with diagnostic uncertainty [3].

Atopic dermatitis (AD) is one of the most common comorbidities of FA during childhood, which may present as immunoglobulin E (IgE)-mediated FA, non-IgE-mediated FA, or a mixture of the two [4]. A large number of children with AD are asymptomatic to specific food allergens but may be sensitized by them [5,6]. This inconsistent association may lead to unnecessary food restrictions in children with moderate and severe AD [5]. When AD is accompanied by FA, clarity regarding the causative food allergen leads to the more accurate and efficient management of AD [3,4,5].

Basophils are thought to be involved closely in a food allergic reaction because their activation correlates with the development of symptoms during an OFC regardless of the tryptase level [7,8]. The basophil activation test (BAT) is a flow cytometry-based assay where the expression of activation markers, such as CD63 and CD203c, present on the surface of basophils, is measured after stimulation with an allergen [9]. CD63 is highly relevant to an IgE-mediated allergic reaction, which is correlated with histamine through intracellular diamine oxidase [10].

There are various studies suggesting a good agreement between the results of BAT and those of OFC [11,12]. In other studies, the performance of BAT in the diagnosis of allergy to different foods has been assessed, including peanut, wheat, cow’s milk, and egg [13,14]. BAT has been suggested to be useful for monitoring the acquisition of oral tolerance to allergenic foods naturally or under immunotherapy [15]. Measurements of basophil sensitivity have been correlated with the threshold of reactivity during an OFC, which can provide information about the severity of the allergic reaction and the risk assessment [15]. However, evaluations of BAT in FA diagnosis remain scarce, and very little information is available concerning the clinical utility of BAT for the diagnosis of FA in children with AD.

In this study, we sought to evaluate the diagnostic performance of the BAT for milk and egg allergy, which are two of the most commonly observed allergies in children [16,17], and to compare it with the serum-specific IgE (sIgE) test, which is generally used for diagnosing FA. We also assessed the utility of the BAT for diagnosing FA in children with AD. Since the BAT has been suggested to assess the severity of an allergic reaction, we assessed the utility of the BAT for predicting the severity of milk and egg allergy by evaluating anaphylaxis.

## 2. Materials and Methods

### 2.1. Study Design

Children who visited the outpatient clinic in Severance Children’s Hospital for a suspected adverse reaction to food from August 2017 to March 2018 were enrolled. Diagnosis of milk or egg allergy was based on a clinical history of repeated allergic symptoms within 2 h after ingestion of egg or milk or a positive OFC. We included patients with IgE-mediated milk or egg allergy with symptoms of the skin and/or mucus membranes (urticaria, increase in eczema, or swollen lips), respiratory tract (throat tightness or shortness of breath, accompanied by signs of bronchus obstruction), or gastrointestinal tract (nausea or vomiting) [2] and whose sIgE levels for milk or egg were >0.1 kU/L [18].

For excluding non-IgE-mediated FA, if allergic symptoms were reported as mild and delayed gastrointestinal (abdominal discomfort, irritable behavior, or bloody stool), the presence of associated symptoms, such as skin symptoms (wheals, rash, or edema), respiratory symptoms, or cardiovascular symptoms, were also required for the diagnosis of FA [19]. We checked the experience of anaphylaxis to milk and egg ingestion according to the 2011 World Allergy Organization Anaphylaxis Guidelines [20]. We simultaneously measured sIgE levels and performed the BAT for cow milk and egg white.

The study was approved by the institutional review board (IRB) of Severance Hospital (Seoul, Korea; IRB no. 4-2017-0514). Written informed consent was obtained from the participants and their parents.

### 2.2. Specific IgE Measurement

We measured sIgE levels for cow’s milk extract and egg white on an ImmunoCAP system (UniCAP^®^, Thermo Fisher Scientific, Waltham, MA, USA) with the appropriate allergen caps according to manufacturer instructions. Milk-specific sIgE values were considered positive if they were >15 kU/L in children aged ≥1 year and 5 kU/L in those aged < 1 year [21]. Egg-specific IgE values were considered positive if they were >7 kU/L in children aged ≥2 years and 2 kU/L in those aged <2 years [21].

### 2.3. The Basophil Activation Test

The BAT was performed with the Flow CAST^®^ kit, dry-powdered cow’s milk extract, and egg white (BÜHLMANN Laboratories AG, Schönenbuch, Switzerland), according to manufacturer instructions. We ensured that no systemically administered antiallergenic medication, such as corticosteroids, were administered for at least 24 h prior to blood sampling [9]. Blood samples were stored at 2–8 °C and analyzed using flow cytometry (FACSCanto II, BD Biosciences, San Jose, CA, USA) within 48 h. Homogenized 50 μL of EDTA whole blood was incubated with stimulation substances, milk or egg extract, with 20 μL of staining reagent (anti-CD63–fluorescein isothiocyanate and anti-CCR3–phycoerythrin mAbs) added, for 15 min at 37 °C in a water bath. After adding 2 mL of lysing reagent, the mixture was incubated for 5 min and centrifuged for 5 min at 500 g. Monoclonal mouse anti-human high-affinity IgE receptor (Anti-FcεRI mAb) and N-formylmethionine leucyl-phenylalanine (fMLP) were used as positive controls. A buffer containing calcium, heparin, and interleukine (IL)-3 was used as a negative control. If both positive controls exhibited activation of >10% of basophils and the negative control exhibited activation of <5% of basophils, allergen stimulation results were regarded as valid. Allergen-induced CD63 and CCR3 expressions were evaluated using flow cytometry (FACSCanto II, BD Biosciences). We adopted more than 15% of basophils with the expression of CCR3 and CD63 as a positive cut-off value according to the manufacturer’s instructions.

### 2.4. Statistical Analysis

Continuous data were reported as the mean (±standard deviation) or median (interquartile range). Comparisons between two groups (patients with milk allergy vs. those without, and patients with egg allergy vs. those without) were performed using Student’s *t*-test for continuous variables and the chi-square test for categorical variables. Spearman’s correlations between sIgE levels and the percentage of basophils with the expression of CD63 were analyzed via Spearman’s rank correlation test. We assessed diagnostic performance using receiver operating characteristic curve analysis, analyzing the area under the curve (AUC). We compared the AUCs of sIgE, the BAT for diagnosis of milk and egg allergy, and for anaphylaxis from milk and egg ingestion using the Delong method [22]. We generated the values that combined the values of sIgE and BAT using a logistic regression model from milk and egg allergy and for anaphylaxis from milk and egg ingestion. The AUCs of sIgE, the BAT, and the combination of sIgE and BAT were also compared using the Delong methods. These comparisons were also performed separately for patients with AD. All statistical analyses were conducted with SAS 9.2 (SAS Institute Inc., Cary, NC, USA). *p* < 0.05 was considered statistically significant.

## 3. Results

### 3.1. Patient Characteristics

Of the 97 children we evaluated, eight were excluded due to their blood samples exhibiting <10% of activated basophils in the positive controls [9]. Among the 89 children included in the study, 14 declined milk-specific IgE measurement, as they had already been drinking milk without any symptoms, and four declined egg-specific IgE measurement for a similar reason. Finally, 75 children were evaluated for milk allergy and 85 for egg allergy in this study. Patient characteristics are summarized in Table 1.

Among the 75 patients who were evaluated for milk allergy, 26 were diagnosed with milk allergy, and, additionally, 33 had AD. Patients with milk allergy were older than those without milk allergy. sIgE levels and the percentage of basophils with the expression of CD63 were higher in patients with milk allergy than in those without milk allergy. Anaphylaxis was experienced by five patients with milk allergy (19.2%).

Among the 85 patients who were evaluated for egg allergy, 42 were diagnosed with egg allergy, and, additionally, 35 had AD. Patients with egg allergy were older than those without egg allergy. sIgE levels and the percentage of basophils with the expression of CD63 were higher in patients with egg allergy than in those without egg allergy. Anaphylaxis was experienced by six patients with egg allergy (14.3%). No significant difference was observed in terms of the proportion of males, the presence of AD, or the presence of a family history of allergy between children with and those without allergy.

Among the 75 patients who were evaluated for milk allergy, 33 were diagnosed with AD. Among the 85 patients who were evaluated for egg allergy, 35 were diagnosed with AD. Patients’ characteristics were compared between total subjects and subjects with AD, and the data are shown in Table 2. There were no significant differences in age, sex, total IgE level, anaphylaxis, and family history. In patients with milk or egg allergy, there were no significant differences in specific IgEs and BAT results for milk or egg between total subjects and subjects with AD.

**Figure 1:**
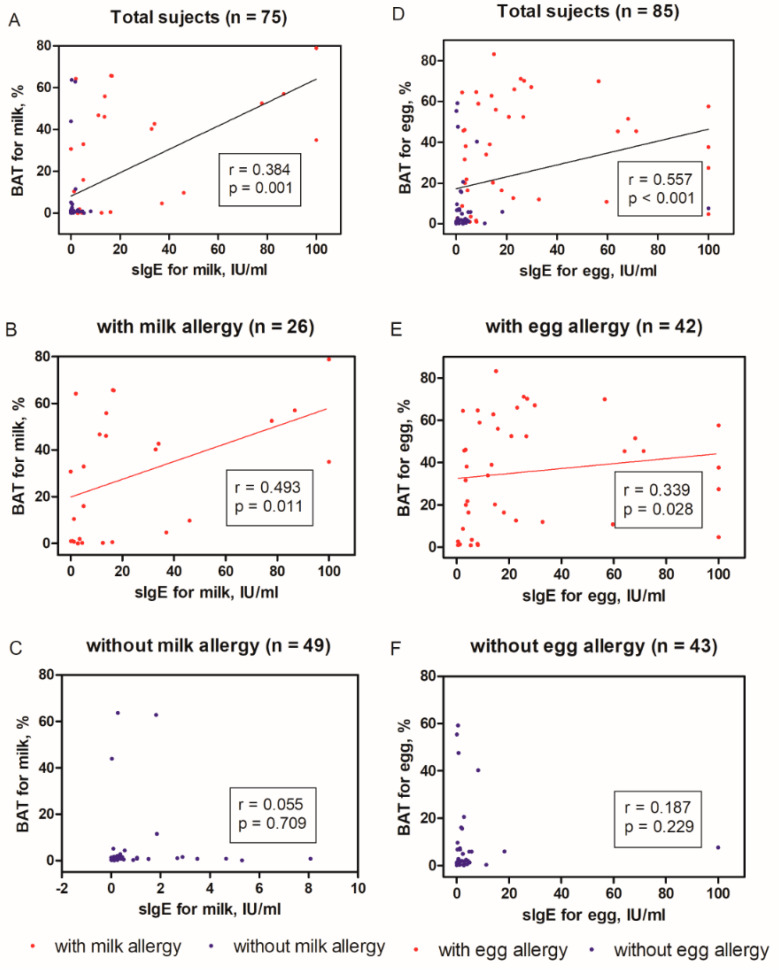
The overall correlation between specific immunoglobulin E (sIgE) levels and percentages of CD63+ basophils using the basophil activation test (BAT), when testing for milk (total subjects shown in (**A**), with milk allergy shown in (**B**), without milk allergy shown in (**C**)) and egg allergy (total subjects shown in (**D**), with milk allergy shown in (**E**), without milk allergy shown in (**F**)). r, Spearman’s correlation coefficient. sIgE, specific immunoglobulin E; BAT, basophil activation test, CD63+ basophils, basophil with the expression of CD63.

There was an overall significant correlation between sIgE level and the percentage of basophils with the expression of CD63 for both milk (r = 0.384, *p* = 0.001) and egg (r = 0.557, *p* < 0.001) allergen in total subjects (Figure 1A,D). The significant correlations between sIgE levels and the percentage of basophils with the expression of CD63 were shown in only patients with milk (r = 0.493, *p* = 0.011) or egg (r = 0.339, *p* = 0.028) allergy, while the correlations were not in the others without milk and egg allergy (Figure 1B,C,E,F).

### 3.2. Diagnostic Performance of the BAT and sIgE

For milk allergy, the AUC was 0.701 for sIgE, 0.758 for the BAT, and 0.805 for a combination of both (Table 3). The AUC for sIgE was significantly increased when combined with the BAT for the diagnosis of milk allergy (*p* = 0.029). For egg allergy, the AUC was 0.777 for sIgE, 0.776 for the BAT, and 0.766 for a combination of both, with no significant difference between any of the above.

We also compared the AUCs for sIgE, the BAT, and a combination of both for patients with anaphylaxis (Table 3). There was no significant difference between any of these.

### 3.3. Diagnostic Performance of the BAT and sIgE for Patients with AD

We compared the AUCs of sIgE, the BAT, and a combination of both for patients with AD (Table 4). Among the 75 patients who were evaluated for milk allergy, 33 patients had AD. Among the 33 patients, nine were diagnosed with a milk allergy. Among the 85 patients who were evaluated for egg allergy, 35 patients had AD. Among the 35 patients, 15 were diagnosed with egg allergy. For milk allergy, the AUCs of the BAT (0.924) and the combination (0.903) were significantly greater (*p* = 0.017 and *p* = 0.026, respectively) than that of sIgE (0.701) in patients with AD. There was no corresponding significant difference for egg allergy.

### 3.4. Distributions of sIgE Levels and Percentages of basophils with the expression of CD63

**Figure 2:**
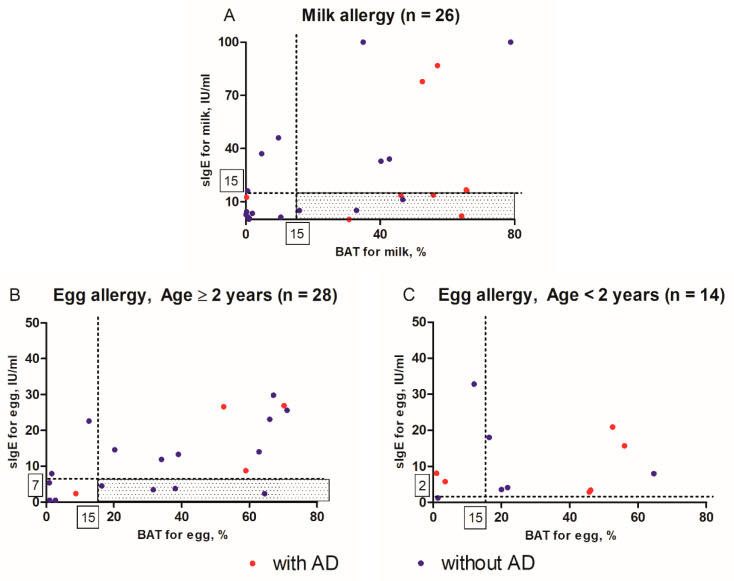
Distributions of specific immunoglobulin E (sIgE) levels and percentages of basophils with the expression of CD63 in (**A**) patients with milk allergy (*n* = 26) and (**B**) patients aged ≥2 years with egg allergy (*n* = 28) and (**C**) patients aged <2 years with egg allergy (*n* = 14). The right lower quadrant (shaded area) represents the area in which a patient with milk or egg allergy tested negative in sIgE and positive in basophil activation test (BAT). Red dots indicate patients with AD, and blue dots indicate without AD. AD, atopic dermatitis; sIgE, specific immunoglobulin E; BAT, basophil activation test.

Plots were constructed for the distributions of sIgE levels and percentages of basophils with the expression of CD63, which are shown in Figure 2, for patients with milk allergy and those with egg allergy. The right lower quadrant (shaded area) represents the area in which a patient with milk or egg allergy tested negative in sIgE but positive in BAT. Among the 26 patients with a milk allergy who were over 1-year-old, seven tested negative in sIgE and positive in BAT. Among them, four had AD, and three had no AD (Figure 2A). Among the 42 patients with egg allergy, 14 were below 2 years of age and had a diagnostic decision point as 2 kU/L of egg sIgE, and the other 28 had a diagnostic decision point as 7 kU/L. Among 28 patients, four had no AD and tested negative in sIgE and positive in BAT (Figure 2B).

## 4. Discussion

In this study, we evaluated the reliability of the BAT as a diagnostic tool for milk and egg allergy in children. The BAT was statistically significantly correlated with sIgE, and the diagnostic performance increased when sIgE was combined with the BAT when testing for milk allergy. When testing for milk allergy in children with AD, the BAT was also demonstrated to be a useful supportive diagnostic tool.

Cow’s milk is the most common and clinically important food allergen in children, as it usually develops during early infancy when cow’s milk is a major nutritional source [23]. However, its diagnosis is often challenging: its symptoms are similar to those of other conditions, including lactose intolerance and food protein-induced enterocolitis syndrome; the reaction differs depending on the temperature to which the milk is heated, and OFC testing can be distressing at such an early age [24,25]. As a large number of children outgrow their milk allergy, they should be serially evaluated for reintroduction [26]. Therefore, an accurate diagnostic tool for milk allergy is needed, and we recommend the BAT as a useful addition.

Classical allergy tests, such as sIgE and skin prick tests, are more difficult to interpret in patients with moderate to severe AD who have higher total IgE levels or are more likely to be sensitized by many different sIgEs [27]. Since a skin prick test or an OFC can result in AD flare-ups, alternatives for the diagnosis of FA in patients with moderate to severe AD are essential [28]. We revealed that the BAT was more useful than sIgE for the diagnosis of milk allergy in children with AD. This may be explained by the nature of the tests: the BAT reflects a functional response induced by cross-linking of FcεRI with the allergen, whereas sIgE assesses only sensitization [29]. A previous study indicated that basophils in AD patients without FA did not spontaneously release elevated levels of histamine, which supports the potential of the BAT for the diagnosis of FA in patients with AD [30].

The BAT has been suggested as an alternative to OFC for predicting the clinical severity of allergic and anaphylactic reactions [31,32]. In our study, the BAT did not provide additional information regarding anaphylaxis to what sIgE did, and the diagnostic reliability of BAT was very low for egg allergy. This may be due to the small number of patients in our study who experienced anaphylaxis, and we did not re-assess OFC-induced anaphylaxis for all patients. The BAT may not be able to predict whether anaphylaxis will occur outside the clinical setting, as there are many contributing factors, such as the allergen dose, comorbidity with other allergic diseases, utilization of medication, and timely and effective treatment [33].

In the past, the BAT was limited due to its direct measurement of histamine level; however, this limitation has been removed, as activation markers are now measured on the surface of basophils [29]. A present limitation of the BAT is that it should preferably be performed within 4 h of sampling for optimal results; however, its viability has been demonstrated up to 24 h after sampling, under optimal storage conditions, and the manufacturer instructions for the reagents we used indicated its use within 48 h of sampling [29,34]. In a hospital with an established laboratory, the clinical use of the BAT is straightforward [35]. Furthermore, the BAT provides a wide variety of allergens for diagnosis, including for various drugs, and can be used to monitor the outcome of oral immunotherapy for FA [9,36]. However, there is a lack of consensus regarding cut-off values for the percentage of basophils with the expression of CD63 and the ideal allergen concentration, and the frequency of non-responders has to be considered (6–17% of the population and 8% of patients in the present study) [9]. Overall, the BAT may be a useful supportive diagnostic tool combined with sensitization tools, including skin prick tests or sIgE, for increasing the diagnostic accuracy of FA and to reduce the need for OFCs.

This study has various limitations that should be considered. First, the number of study participants was small, especially those who had undergone an OFC, and most diagnoses were based on previous allergic reactions. Therefore, we could not assess the severity of allergic symptoms, as these depend on, for example, the degree of baking and amount of milk or egg consumed [31]. Second, the cut-off values of sIgE level for the diagnosis of milk and egg allergy have been reported to be different based on racial and ethnic disparity [37]. Since we did not have the cut-off values for sIgE, which were representative of our regional and ethnic population in diagnosing FA, we used the cut-off values previously suggested in other studies [21,38]. We also could not make recommendations with regard to cut-off values for the BAT, especially for patients experiencing OFC-induced anaphylaxis, due to the small sample size. However, we suggest herein that the BAT may be of particular utility for the diagnosis of specific FA, such as for milk, and for patients with specific comorbidities, such as AD.

In conclusion, BAT may be a supportive diagnostic tool, combined with serum sIgE, for milk allergy in children. It may be especially useful in children with AD.

## Figures and Tables

**Table 1:** Subjects’ characteristics.

	Tested for Milk Allergy (*n* = 75)		Tested for Egg Allergy (*n* = 85)	
	Children with Milk Allergy (*n* = 26)	Children without Milk Allergy (*n* = 49)	Children with Egg Allergy (*n* = 42)	Children without Egg Allergy (*n* = 43)
Age, year	3.0 (2.0–5.2) *	1.6 (0.9–2.8)	2.6 (1.4–5.3) ^†^	1.8 (0.9–2.5)
Male, *n* (%)	15 (57.7)	30 (61.2)	28 (66.7)	23 (53.5)
Total IgE, kU/L	557 (277–1210) *	90 (37–280)	296 (81–907) ^†^	99 (33–322)
Specific IgE for milk or egg, kU/L	13.10 (2.61–34.85) *	0.19 (0.09–0.72)	14.30 (4.01–30.55) ^†^	1.09 (0.27–3.20)
CD63+ basophils for milk or egg, %	31.8 (1.0–53.3) *	1.0 (0.5–1.5)	37.9 (11.7–57.9) ^†^	1.9 (0.8–6.8)
Anaphylaxis, *n* (%)	5 (19.2) *	0 (0)	6 (14.3) ^†^	0 (0)
Atopic dermatitis, *n* (%)	9 (34.6)	24 (49.0)	15 (35.7)	20 (46.5)
Family history of allergy, *n* (%)	7 (26.9)	18 (36.7)	15 (35.7)	14 (32.6)
Other suspected allergic foods ^‡^				
Nuts, *n* (%)	6 (23.1)	7 (14.3)	5 (11.9)	5 (11.6)
Soybean, *n* (%)	1 (3.8)	2 (4.1)	1 (2.4)	2 (4.7)
Wheat, *n* (%)	4 (15.4)	4 (8.2)	6 (14.3)	3 (7.0)
Fruits and vegetables, *n* (%)	2 (7.7)	14 (28.6)	10 (23.8)	7 (16.3)
Fish, *n* (%)	2 (7.7)	4 (8.2)	4 (9.5)	3 (7.0)
Crustaceans, *n* (%)	1 (3.8)	4 (8.2)	2 (4.8)	3 (7.0)

IgE, immunoglobulin E, CD63+ basophils, basophil with the expression of CD63. Age, IgE levels, and CD63+ basophil levels are indicated as the median (interquartile range). * *p* < 0.05 vs. without milk allergy, ^†^ *p* < 0.05 vs. without egg allergy. ^‡^ Other suspected allergic foods were reported from the children’s parents.

**Table 2:** Comparison of subjects’ characteristics between total subjects and subjects with AD.

	Tested for Milk Allergy (*n* = 75)		Tested for Egg Allergy (*n* = 85)	
	**All (*n* = 75)**	**with AD (*n* = 33)**	**All (*n* = 85)**	**with AD (*n* = 35)**
Age, year	2.1 (1.1–4.6)	1.6 (0.8–4.4)	2.1 (1.1–3.6)	1.6 (0.8–2.6)
Male, *n* (%)	45 (60.0)	19 (57.6)	51 (60.0)	21 (60.0)
Total IgE, kU/L	156 (48–655)	113 (52–706)	155 (52–604)	99 (44–557)
Anaphylaxis, *n* (%)	5 (6.7)	3 (9.1)	6 (7.1)	1 (2.9)
Family history of allergy, *n* (%)	25 (33.3)	8 (24.2)	29 (34.1)	10 (28.6)
Milk or egg allergy, *n* (%)	26 (34.7)	9 (27.3)	42 (49.4)	15 (42.9)
Specific IgE for milk or egg in milk or egg allergy, kU/L	13.1 (2.6–34.9)	13.8 (7.2–47.3)	14.3 (4.0–30.6)	15.7 (5.8–64.1)
CD63+ basophils for milk or egg in milk or egg allergy, %	31.8 (0.1–53.3)	55.8 (38.4–64.9)	37.9 (11.7–57.9)	51.5 (45.4–57.6)

AD, atopic dermatitis, IgE, immunoglobulin E, CD63+ basophils, basophil with the expression of CD63. Age and IgE levels are indicated as the median (interquartile range).

**Table 3:** Diagnostic performance of allergy tests in milk and egg allergy and anaphylaxis for total subjects.

	Diagnosis	Diagnostic Tool	AUC (95% CI)	Comparison of AUCs between (*p*-Value)		
				sIgE vs. BAT	BAT vs. Combined	sIgE vs. Combined
Tested for milk allergy (*n* = 75)	Milk allergy (*n* = 26)	sIgE	0.701 (0.602–0.800)	0.373	0.157	0.029 *
		BAT	0.758 (0.655–0.860)			
		Combined sIgE and BAT	0.805 (0.707–0.904)			
	Anaphylaxis (*n* = 5)	sIgE	0.790 (0.651–0.929)	0.949	0.784	0.991
		BAT	0.797 (0.532–1.000)			
		Combined sIgE and BAT	0.791 (0.509–1.000)			
Tested for egg allergy (*n* = 85)	Egg allergy (*n* = 42)	sIgE	0.777 (0.687–0.866)	0.989	0.830	0.781
		BAT	0.776 (0.687–0.865)			
		Combined sIgE and BAT	0.766 (0.681–0.852)			
	Anaphylaxis (*n* = 6)	sIgE	0.624 (0.476–0.772)	0.661	0.867	0.705
		BAT	0.577 (0.367–0.787)			
		Combined sIgE and BAT	0.561 (0.304–0.818)			

sIgE, specific immunoglobulin E; BAT, basophil activation test; AUC, area under the receiver operating characteristic curve; CI, confidence interval; Combined, Combined sIgE and BAT. * *p* < 0.05 for comparison of AUCs using the Delong method.

**Table 4:** Diagnostic performance of allergy tests in milk and egg allergy for patients with AD.

	Diagnosis	Diagnostic Tool	AUC (95% CI)	Comparison of AUCs between (*p*-Value)		
				sIgE vs. BAT	BAT vs. Combined	sIgE vs. Combined
Tested for milk allergy (*n* = 33)	Milk allergy (*n* = 9)	sIgE	0.701 (0.524–0.878)	0.017 *	0.317	0.026 *
		BAT	0.924 (0.807–1.000)			
		Combined sIgE and BAT	0.903 (0.780–1.000)			
Tested for egg allergy (*n* = 35)	Egg allergy (*n* = 15)	sIgE	0.850 (0.747–0.953)	0.768	0.712	0.146
		BAT	0.825 (0.693–0.957)			
		Combined sIgE and BAT	0.800 (0.690–0.910)			

AD, atopic dermatitis; sIgE, specific immunoglobulin E; BAT, basophil activation test; AUC, area under the receiver operating characteristic curve. * *p* < 0.05 for comparison of AUCs using the Delong method.

## References

[1] Cox A.L., Nowak-Wegrzyn A. (2018). Innovation in food challenge tests for food allergy. Curr. Allergy Asthma Rep..

[2] Gupta M., Cox A., Nowak-Węgrzyn A., Wang J. (2018). Diagnosis of food allergy. Immunol. Allergy Clin. N. Am..

[3] Sicherer S.H., Sampson H.A. (2018). Food allergy: A review and update on epidemiology, pathogenesis, diagnosis, prevention, and management. J. Allergy Clin. Immunol..

[4] Cartledge N., Chan S. (2018). Atopic dermatitis and food allergy: A paediatric approach. Curr. Pediatr. Rev..

[5] Graham F., Eigenmann P.A. (2020). Atopic dermatitis and its relation to food allergy. Curr. Opin. Allergy Clin. Immunol..

[6] Broeks S.A., Brand P.L. (2017). Atopic dermatitis is associated with a fivefold increased risk of polysensitisation in children. Acta Paediatr.

[7] Sampson H.A., Mendelson L., Rosen J.P. (1992). Fatal and near-fatal anaphylactic reactions to food in children and adolescents. N. Engl. J. Med..

[8] Commins S.P., James H.R., Stevens W., Pochan S.L., Land M.H., King C. (2014). Delayed clinical and ex vivo response to mammalian meat in patients with IgE to galactose-alpha-1,3-galactose. J. Allergy Clin. Immunol..

[9] Hoffmann H.J., Santos A.F., Mayorga C., Nopp A., Eberlein B., Ferrer M., Rouzaire P., Ebo D.G., Sabato V., Sanz M.L. (2015). The clinical utility of basophil activation testing in diagnosis and monitoring of allergic disease. Allergy.

[10] Ebo D.G., Bridts C.H., Mertens C.H., Hagendorens M.M., Stevens W.J., De Clerck L.S. (2012). Analyzing histamine release by flow cytometry (HistaFlow): A novel instrument to study the degranulation patterns of basophils. J. Immunol. Methods.

[11] Ruinemans-Koerts J., Schmidt-Hieltjes Y., Jansen A., Savelkoul H.F.J., Plaisier A., van Setten P. (2019). The basophil activation test reduces the need for a food challenge test in children suspected of ige-mediated cow’s milk allergy. Clin. Exp. Allergy.

[12] Ocmant A., Mulier S., Hanssens L., Goldman M., Casimir G., Mascart F., Schandene L. (2009). Basophil activation tests for the diagnosis of food allergy in children. Clin. Exp. Allergy.

[13] Sato S., Tachimoto H., Shukuya A., Kurosaka N., Yanagida N., Utsunomiya T., Iguchi M., Komata T., Imai T., Tomikawa M. (2010). Basophil activation marker cd203c is useful in the diagnosis of hen’s egg and cow’s milk allergies in children. Int. Arch. Allergy Immunol..

[14] Sato S., Yanagida N., Ebisawa M. (2018). How to diagnose food allergy. Curr. Opin. Allergy Clin. Immunol..

[15] Santos A.F., Shreffler W.G. (2017). Road map for the clinical application of the basophil activation test in food allergy. Clin. Exp. Allergy.

[16] Savage J., Johns C.B. (2015). Food allergy: Epidemiology and natural history. Immunol. Allergy Clin. N. Am..

[17] Lee S.Y., Ahn K., Kim J., Jang G.C., Min T.K., Yang H.J., Pyun B.Y., Kwon J.W., Sohn M.H., Kim K.W. (2016). A multicenter retrospective case study of anaphylaxis triggers by age in korean children. Allergy Asthma Immunol. Res..

[18] Scala G., Miceli Sopo S. (2015). When are serum specific ige levels positive?. J. Allergy Clin. Immunol..

[19] Nowak-Wegrzyn A., Katz Y., Mehr S.S., Koletzko S. (2015). Non-ige-mediated gastrointestinal food allergy. J. Allergy Clin. Immunol..

[20] Simons F.E., Ardusso L.R., Bilo M.B., El-Gamal Y.M., Ledford D.K., Ring J., Sanchez-Borges M., Senna G.E., Sheikh A., Thong B.Y. (2011). World allergy organization anaphylaxis guidelines: Summary. J. Allergy Clin. Immunol..

[21] Burks A.W., Holgate S.T., O’Hehir R.E., Bacharier L.B., Broide D.H., Hershey G.K.K., Peebles R.S. (2019). Middleton’s Allergy e-Book: Principles and Practice.

[22] DeLong E.R., DeLong D.M., Clarke-Pearson D.L. (1988). Comparing the areas under two or more correlated receiver operating characteristic curves: A nonparametric approach. Biometrics.

[23] Mousan G., Kamat D. (2016). Cow’s milk protein allergy. Clin. Pediatr. (Phila.).

[24] Greiwe J. (2019). Oral food challenges in infants and toddlers. Immunol. Allergy Clin. N. Am..

[25] Agyemang A., Nowak-Wegrzyn A. (2019). Food protein-induced enterocolitis syndrome: A comprehensive review. Clin. Rev. Allergy Immunol..

[26] Luyt D., Ball H., Makwana N., Green M.R., Bravin K., Nasser S.M., Clark A.T. (2014). Bsaci guideline for the diagnosis and management of cow’s milk allergy. Clin. Exp. Allergy.

[27] Du Toit G., Santos A., Roberts G., Fox A.T., Smith P., Lack G. (2009). The diagnosis of ige-mediated food allergy in childhood. Pediatr Allergy Immunol..

[28] Nguyen T.A., Leonard S.A., Eichenfield L.F. (2015). An update on pediatric atopic dermatitis and food allergies. J. Pediatr..

[29] Hemmings O., Kwok M., McKendry R., Santos A.F. (2018). Basophil activation test: Old and new applications in allergy. Curr. Allergy Asthma Rep..

[30] Sampson H.A., Broadbent K.R., Bernhisel-Broadbent J. (1989). Spontaneous release of histamine from basophils and histamine-releasing factor in patients with atopic dermatitis and food hypersensitivity. N. Engl. J. Med..

[31] Ford L.S., Bloom K.A., Nowak-Wegrzyn A.H., Shreffler W.G., Masilamani M., Sampson H.A. (2013). Basophil reactivity, wheal size, and immunoglobulin levels distinguish degrees of cow’s milk tolerance. J. Allergy Clin. Immunol..

[32] Song Y., Wang J., Leung N., Wang L.X., Lisann L., Sicherer S.H., Scurlock A.M., Pesek R., Perry T.T., Jones S.M. (2015). Correlations between basophil activation, allergen-specific ige with outcome and severity of oral food challenges. Ann. Allergy Asthma Immunol..

[33] Smith P.K., Hourihane J.O., Lieberman P. (2015). Risk multipliers for severe food anaphylaxis. World Allergy Organ. J..

[34] Flow CAST^®^: Basophil Activation Test, Flow Cytometry [Internet] Schönenbuch, Switzerland: BUHLMANN LABORATORIES AG. https://www.buhlmannlabs.ch/products-solutions/cellular-allergy/flow-cast/.

[35] Depince-Berger A.E., Sidi-Yahya K., Jeraiby M., Lambert C. (2017). Basophil activation test: Implementation and standardization between systems and between instruments. Cytometry A.

[36] De Amici M., Barocci F., Caimmi S., Nespoli L., Licari A., Giuliani G., Marseglia G. (2019). Clinical use of basophil activation test in drug, food and hymenoptera venom allergies. Minerva Pediatr..

[37] Greenhawt M., Weiss C., Conte M.L., Doucet M., Engler A., Camargo C.A. (2013). Racial and ethnic disparity in food allergy in the United States: A systematic review. J. Allergy Clin. Immunol. Pract..

[38] Sampson H.A. (2001). Utility of food-specific IgE concentrations in predicting symptomatic food allergy. J. Allergy Clin. Immunol..

